# Three New Isoprenylated Flavones from *Artocarpus chama* Stem and Their Bioactivities

**DOI:** 10.3390/molecules27010003

**Published:** 2021-12-21

**Authors:** Sukanya Dej-adisai, Kedsaraporn Parndaeng, Chatchai Wattanapiromsakul, Jae Sung Hwang

**Affiliations:** 1 Department of Pharmacognosy and Pharmaceutical Botany, Faculty of Pharmaceutical Sciences, Prince of Songkla University, Hat Yai, Songkhla 90112, Thailand; kedsarapon.p@gmail.com (K.P.); chatchai.w@psu.ac.th (C.W.); 2 Department of Genetic Engineering & Graduate School of Biotechnology, College of Life Sciences, Kyung Hee University, Youngin 17104, Korea; jshwang@khu.ac.kr

**Keywords:** *Artocarpus charma*, moraceae, isoprenylated flavone, antityrosinase activity, melanin content, antimicrobial activity

## Abstract

Phytochemical investigation of *Artocarpus chama* stem was performed by chromatographic techniques, resulting from the isolation and structure elucidation of three new compounds, namely 3′-farnesyl-apigenin (**1**), 3-(hydroxyprenyl) isoetin (**2**), and 3-prenyl-5,7,2′,5′-tetrahydroxy-4′-methoxyflavone (**3**), and five known compounds, namely homoeriodictyol (**4**), isocycloartobilo-xanthone (**5**), artocarpanone (**6**), naringenin (**7**), and artocarpin (**8**). From the screening result, *A. chama* extract showed a potent tyrosinase inhibitory effect. Ihe isolated compounds 1, 4 and 6 also exhibited tyrosinase inhibition with IC_50_ of 135.70, 52.18, and 38.78 µg/mL, respectively. Moreover, compounds **3**, **4**, **5**, **6**, and **8** showed strong activity against *Staphylococcus epidermidis*, *S. aureus*, methicillin-resistant *S. aureus*, and *Cutibacterium acnes*. This study is the first report on phytochemical investigation with new compounds and biological activities of *A. chama*. Skin infection can cause dark spots or hyperpigmentation. The isolated compounds that showed both anityrosinase and antimicrobial activities will be further studied in in vivo and clinical trials in order to develop treatment for hyperpigmentation, which is caused by infectious diseases by microorganisms.

## 1. Introduction

Nowadays, skin whitening, or skin lightening, is popular; thus, skin-whitening agents have been searching. Melanin is an important factor that determines skin color [1]. Melanin is a polyphenolic pigment and causes dark-colored skin. It is produced in the process called melanogenesis [2]. In melanogenesis, tyrosinase enzymes, an important enzyme, catalyzes l-tyrosine to l-Dopa and to *o*-Dopaquinone-H^+^ before passing the intermediate to the final melanin [3].

Tyrosinase is one of the main enzymes in the melanogenesis process. Therefore, inhibition of tyrosinase activity can decrease melanogenesis. The most commonly used treatment for all types of hyperpigmentary disorders is topical hydroquinone. It can lead side-effect reactions, such as skin irritation, dermatitis, melanocyte demolition, ochronosis, post-inflammatory pigmentation, cytotoxicity, and skin cancer [4].

Tyrosinase inhibitors from natural product might be an alternative way to provide the compounds for antityrosinase activity because plants are a rich source of bioactive chemicals that are mostly free from harmful side effects [5]. Nowadays, interest in tyrosinase inhibitors from natural sources is increasing [6,7,8,9,10]. Moraceae is the most interesting plant family to study concerning antityrosinase activity because many compounds from this family showed inhibitory activity against tyrosinase enzymes [11]. *Artocarous chama*, which is a plant in the Moraceae family, has been reported to show antioxidant activity [12,13] and was screened for antityrosinase and antibacterial activities [11], as well as chemical constituents, mostly flavonoids and phenolic compounds [14,15,16]. Flavonoids play a key role in newly discovered natural tyrosinase inhibitors [17,18]. The number and location of phenolic hydroxyl groups on flavonoids significantly influences the inhibition of tyrosinase activity [19,20,21].

Infection by pathogenic bacteria, for example, *Cutibacterium acnes*, *Staphylococcus epidermidis*, and *S. aureus*, can cause acne vulgaris or acne inflammation resulting in post-inflammatory hyperpigmentation [22,23]. Therefore, effective treatment of hyperpigmentation should include antimicrobial agents.

Tyrosinase inhibition is important to reduction of melanin, may be developed into new drug to treatments for hyperpigmentation [24], and could be useful in cosmetology [25]. Tyrosinase inhibitors from natural products are getting a lot of attention. This is because plant-based tyrosinase inhibitors are cost-effective and cause fewer side effects.

## 2. Results

### 2.1. Structure Elucidation of Isolated Compounds

**Figure 1:**
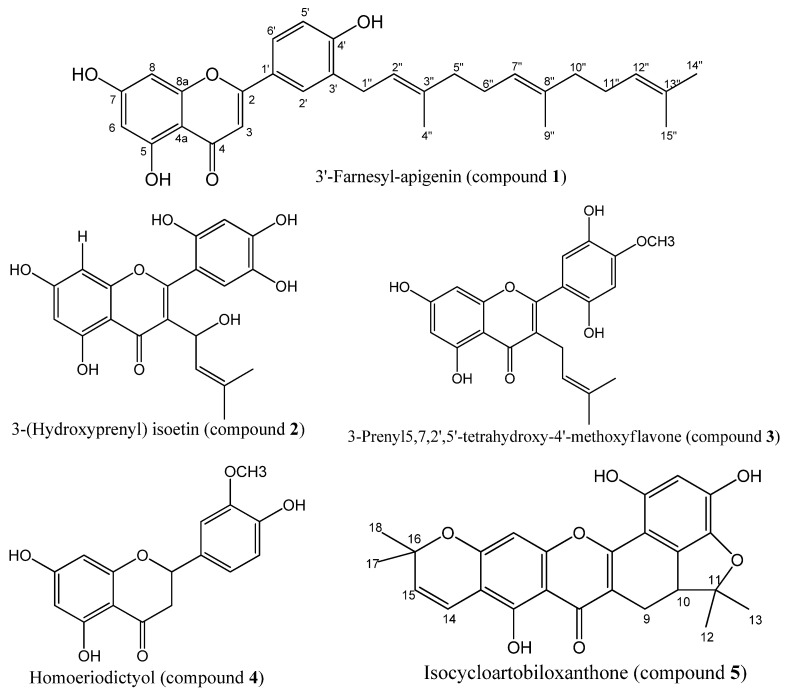

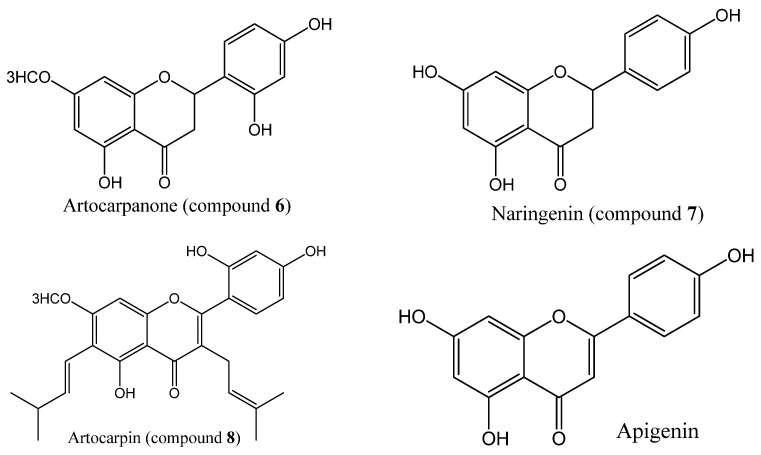
The isolated compounds and apigenin.

Eight pure compounds from EtOAc extract of *A. chama* stem were isolated and identified by using physical properties and spectroscopic data. Then, their chemical structures were confirmed, by comparison with previous reports, as 3′-Farnesylapigenin (Compound **1**), 3-(Hydroxyprenyl) isoetin (Compound **2**), 3-Prenyl-5,7,2′,5′- tetrahydroxy-4′-methoxyflavone (Compound **3**), Homoeriodictyol (Compound **4**), Isocycloartobiloxanthone (Compound **5**), Artocarpanone (Compound **6**), Naringenin (Compound **7**), and artocarpin (Compound **8**) (Figure 1).

#### 2.1.1. 3′-Farnesyl Apigenin; (Compound **1**)

**Figure 2:**
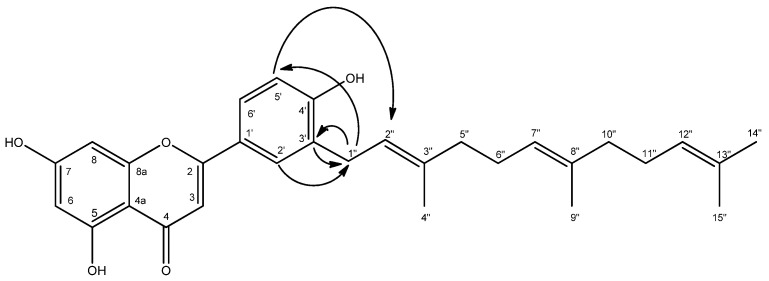
HMBC correlations (from H to C) of compound **1**.

Compound **1** was obtained as a yellow, amorphous powder, soluble in methanol. The UV spectrum in chloroform demonstrated an absorption maximum at λ_max_ 228, 242, 286, and 396 nm. The IR spectrum exhibited maximum absorption bands at 3401, 2923, 2851, and 1622 cm^−1^. The HR-EIMS, which showed a molecular ion peak at *m*/*z* 497.2298 calcd., confirmed a molecular formula of C_30_H_34_O_5_, 497.2298. The ^1^H-NMR spectrum exhibited three olefinic proton signals of typical ortho and meta-coupled patterns of ring B at δ_H_ 6.87 (1H, d, *J* = 8.2 Hz, H-5′), 7.68 (1H, dd, *J* = 8.2, 2.4 Hz, H-6′), and 7.63 (1H, d, *J* = 2.4 Hz, H-2′). Two olefinic proton signals of a typical meta-coupled pattern due to a 1,2,3,5-tetrasubstituted benzene ring A at δ_H_ 6.18 (1H, d, *J* = 2.2 Hz, H-6) and 6.39 (1H, d, *J* = 2.1 Hz, H-8). The olefinic proton signal at δ_H_ 6.49 (1H, s, H-3) indicates the characteristic of flavone-ring C structure. The farnesyl moiety proton signals: three olefinic protons at δ_H_ 5.34 (1H, td, *J* = 7.3, 1.2 Hz, H-2″), 5.10 (1H, td, *J* = 7.0, 1.2 Hz, H-7″), and 4.94 (1H, dd, *J* = 7.0, 1.4 Hz, H-12″); five methylenes at δ_H_ 3.35 (2H, d, *J* = 7.3 Hz, H-1″), 2.14 (2H, dd, *J* = 28.7, 6.5 Hz, H-6″), 2.08 (2H, dd, *J* = 28.5, 6.5 Hz, H-5″), 1.92 (2H, dd, *J* = 33.4, 7.0 Hz, H-11″), and 1.85 (2H, dd, *J* = 32.7, 7.0 Hz, H-10″); and four methyls at δ_H_ 1.73 (3H, d, *J* = 1.3 Hz, H-4″), 1.56 (3H, d, *J* = 1.4 Hz, H-14″), 1.54 (3H, d, *J* = 1.3 Hz, H-9″), and 1.48 (3H, d, *J* = 1.3 Hz, H-15″). The abovementioned evidence suggests that compound **1** is a trihydroxyflavone compound with a farnesyl group. The ^13^C-NMR spectrum showed 30 carbon signals. The trihydroxyflavone moiety showed: one conjugated ketone signal at δ_C_ 183.83 (C-4), five oxygenated olefinic quaternary signals at δ_C_ 166.57 (C-2), 165.93 (C-7), 163.19 (C-5), 159.39 (C-8a), and 160.50 (C-4′); three olefinic quaternary signals at δ_C_ 130.37 (C-3′), 123.10 (C-1′), and 105.33 (C-4a); and six olefinic methine signals at δ_C_ 128.92 (C-2′), 126.86 (C-6′), 116.25 (C-5′), 103.68 (C-3), 100.10 (C-6), and 95.05 (C-8). In addition, the following signals of a farnesyl moiety, confirmed with previous report [26], were observed: three olefinic quaternary signals at δ_C_ 137.44 (C-3″), 136.15 (C-8″), and 131.96 (C-13″); two olefinic methines at δ_C_ 125.40 (C-12″), 125.14 (C-7″), and 123.48 (C-2″); five methylenes at δ_C_ 40.80 (C-10″), 40.72 (C-5″), 28.93 (C-1″), 27.77 (C-11″), and 27.37 (C-6″); and four methyls at δ_C_ 25.81 (C-14″), 17.66 (C-15″), 16.26 (C-4″), and 16.16 (C-9″). The flavone structure and the location of the farnesyl group were proven by HMBC NMR experiments. The HMBC experiment suggested the substitution of the farnesyl moiety could be formed at the C-3′ of ring B, which was confirmed by the key correlations in the HMBC spectrum (Figure 2). Therefore, the structure of compound **1** was elucidated as 3′-farnesyl apigenin, a new compound.

#### 2.1.2. 3-(Hydroxyprenyl) Isoetin; (Compound **2**)

**Figure 3:**
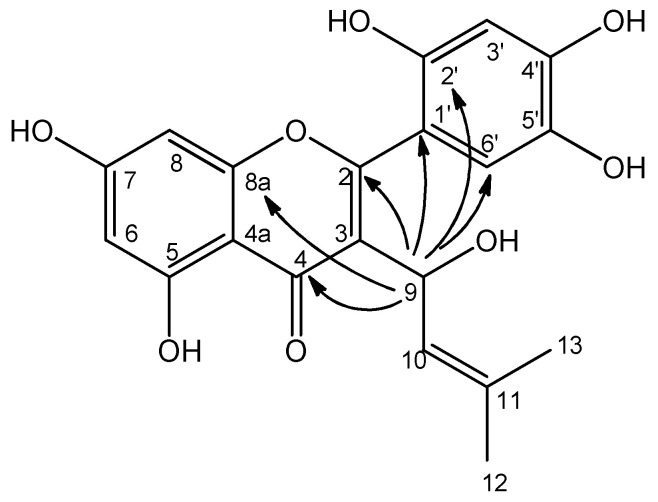
HMBC correlations (from H to C) of compound **2**.

Compound **2** was obtained as a yellow, amorphous powder, soluble in methanol. The UV spectrum in methanol demonstrated a maximum absorption at λ_max_ 237, 275, and 292 nm. The IR spectrum exhibited maximum absorption bands at 3435, 1652, and 1550 cm^−1^. The ESIMS showed an [M + Na]^+^ ion peak at *m*/*z* 409.1618, correlated with a molecular formula of C_20_H_18_O_8_. In the ^1^H-NMR spectrum, two olefinic signals at δ_H_ 7.13 (1H, s, H-6′) and 6.33 (1H, s, H-3′), owing to a tetrasubstituted benzene ring, B, and two olefinic signals of a typical meta-coupled pattern at δ_H_ 6.37 (1H, d, *J* = 1.7 Hz, H-8) and 6.16 (1H, d, *J* = 1.9, Hz, H-6), due to a 1,2,3,5-tetrasubstituted benzene ring, A, were observed. In addition, hydroxyprenyl moiety proton signals of two methines at δ_H_ 6.09 (1H, d, *J* = 9.2 Hz, H-9) and 5.42 (1H, dt, *J* = 9.2, 1.2 Hz, H-10) were observed. The abovementioned evidence suggests that compound **2** is a pentahydroxyflavone compound with a hydroxyprenyl group. The oxygenated carbon signals at δ_C_ 70.21 indicate a hydroxyl group of the side chain, hydroxyprenyl. The flavone structure and the location of hydroxyprenyl moiety at C-3 were confirmed based on the HMBC experiments (Figure 3). Thus, the structure of compound **2** was determined to be 3-(hydroxyprenyl) isoetin, a new compound.

#### 2.1.3. 3-Prenyl-5,7,2′,5′-Tetrahydroxy-4′-Methoxyflavone; (Compound **3**)

**Figure 4:**
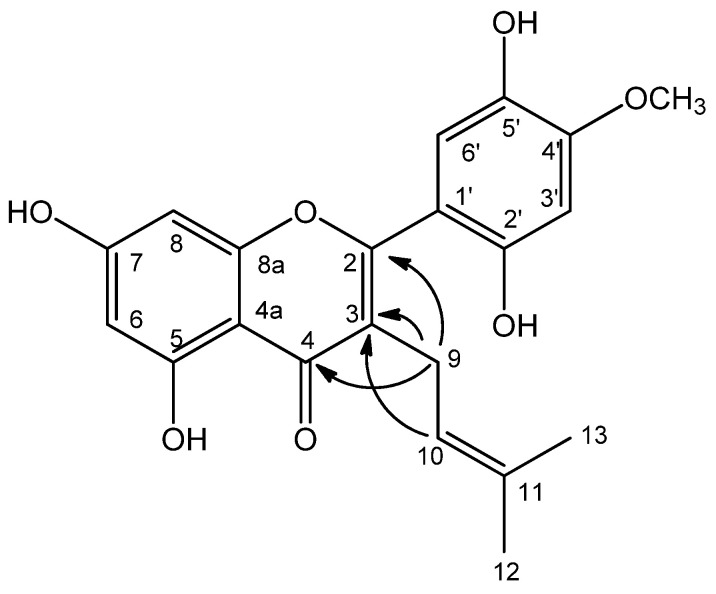
HMBC correlations (from H to C) of compound **3**.

Compound **3** was obtained as a yellow, amorphous powder, soluble in methanol. The UV spectrum in methanol demonstrated a maximum absorption at λ_max_ 205, 253, and 331 nm. The IR spectrum exhibited maximum absorption bands at 3412, 1652, 1615, 1512, 1448, 1430, 1359, 1297, and 1166 cm^−1^. The HR-ESIMS showed an [M + Na]^+^ ion peak at *m*/*z* 407.1101, correlated with a molecular formula of C_21_H_20_O_7_. The ^1^H-NMR and ^13^C-NMR data of compound **3** were quite similar to those of compound **2**, except for the replacement of the methylene signal at δ_H_ 3.01 (2H, d, *J* = 7.0 Hz, H-9) and the methoxyl signal at δ_H_ 3.71 (3H, s, 4′-OCH_3_) of ring B. The carbon signals of compound **3** were in agreement with those of compound **2**, except that the carbon signal at position C-9 (24.85) was shifted up field by 45.36 ppm, compared with the carbon chemical shift of compound **2** (70.21), due to oxygenation effects, indicating a methylene group at C-9. Moreover, the signals of the B ring suggested that the methoxy group in compound **3** was possibly presented in the B ring. These findings indicated that compound **3** is was a tetrahydroxyflavone compound with a prenyl group. The flavone structure and the location of the prenyl moiety were confirmed based on the HMBC experiment (Figure 4). Thus, the structure of compound **3** was determined to be 3-prenyl-5,7,2′,5′-tetrahydroxy- 4′- methoxyflavone, a new prenylated flavone.

### 2.2. Enzymatic Antityrosinase Activity

Tyrosinase inhibitory activity of the isolated compounds from *A. chama* was determined by the dopachrome method. Artocarpanone (Compound **6**), showed the highest activity against tyrosinase enzymes, with IC_50_ of 38.78 μg/mL, followed by homoeriodictyol (Compound **4**), with IC_50_ of 52.18 μg/mL, while, 3′-farnesyl-apigenin (Compound **1**), exhibited the moderate tyrosinase inhibitory activity, with IC_50_ of 135.70 μg/mL. Five isolated compounds showed IC_50_ of higher than 200 μg/mL: isocycloartobiloxanthone (Compound **5**), 3-(hydroxyprenyl) isoetin (Compound **2**), 3-prenyl-5,7,2′,5′-tetrahydroxy-4′-methoxyflavone (Compound **3**), naringenin (Compound **7**), and artocarpin (Compound **8**). The results are presented in Table 1.

### 2.3. Cell Viability

**Figure 5:**
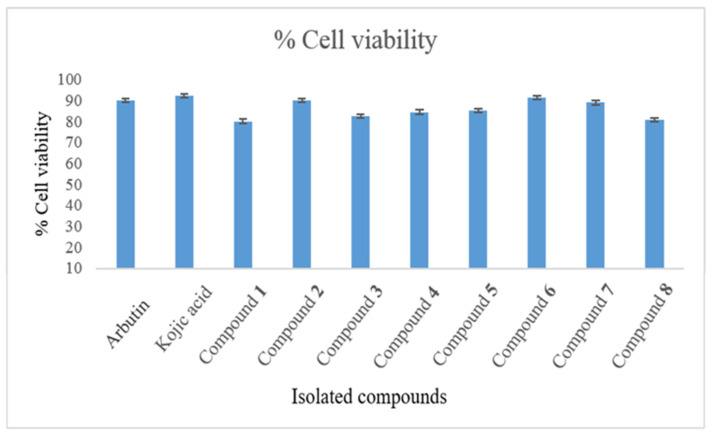
Cell viability of isolated compounds at a concentration 50 μg/mL.

The initial purpose was to test whether the isolated compounds could inhibit melanogenesis in cultured melanocytes without affecting cell growth. The results showed that all the isolated compounds were not toxic to B16-F1 melanoma cells. Cell viability was still more than 80% at the highest concentration of 50 μg/mL. The results are presented in Figure 5.

### 2.4. Intracellular Antityrosinase Activity and Melanin Content

**Figure 6:**
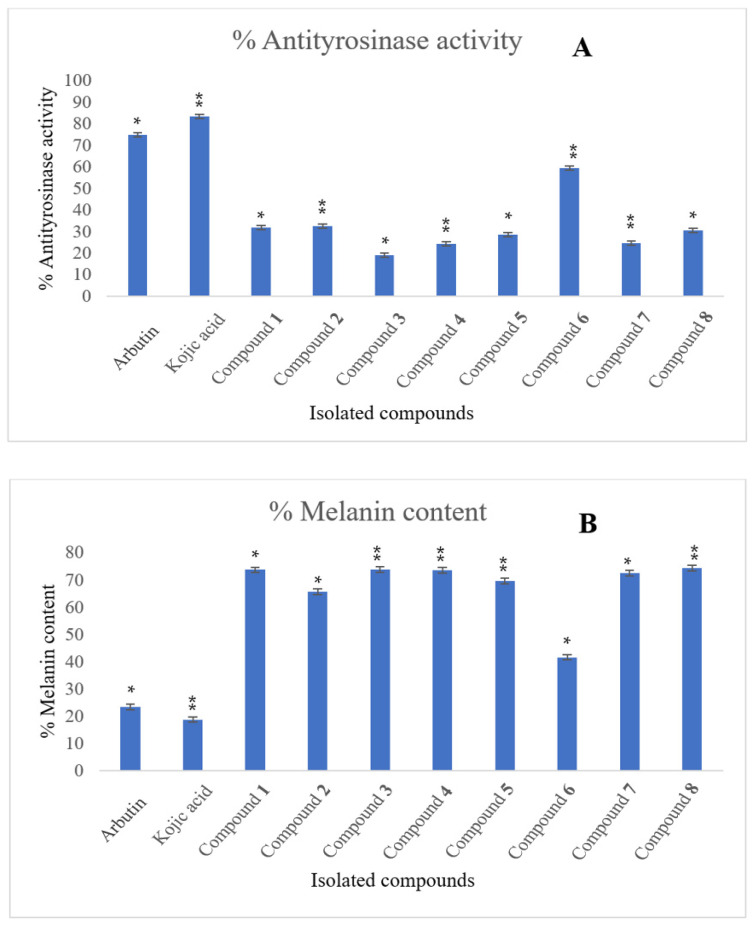
Intracellular antityrosinase activity (**A**) and melanin content (**B**). Data were expressed as mean ± SD from three independent experiments. * *p* < 0.05, and ** *p* < 0.01 indicated a significant difference from the positive control group. Positive controls were arbutin and kojic acid.

The effects of the isolated compounds at concentrations of 50 µg/mL on intracellular antityrosinase activity and melanin content of B16F1 melanoma cells was determined. The antityrosinase activity of supernatants was measured, and the results showed that Compound **6** (artocarpanone) exhibited antityrosinase activity (Figure 6A). Moreover, melanin content showed an inverse result with antityrosinase activity; usually the increase of antityrosinase activity led to the decrease of melanin content (Figure 6B).

### 2.5. Antimicrobial Activity Assay

From the screening of antimicrobial activity [11], ethyl acetate extracts of *A. chama* and *S. taxoides* showed potential effects against *S. epidermidis*, *S. aureus*, MRSA, and *P. acnes.* Thus, the ethyl acetate extracts of these plants were selected for further isolation of active compounds. Then, the MIC and MBC of isolated compounds were investigated for *S. epidermidis*, *S. aureus*, MRSA, and *C. acnes.* The MIC and MBC values of the isolated compounds are presented in Table 2.

## 3. Discussion

All isolated compounds (Figure 1) were in the flavonoid group and can be divided in two sub-groups. The group first is flavanones, composed of homoeriodictyol, artocarpanone, and naringenin, while the second group is flavones, composed of 3′-farnesyl-apigenin, 3-(hydroxylprenyl) isoetin, 3-prenyl-5,7,2′,5′-tetrahydroxy-4′-methoxyflavone, isocycloartobiloxanthone, and artocarpin. Homoeriodictyol, artocarpanone, and 3′-farnesyl-apigenin showed direct activity against tyrosinase enzymes. The related 4-substituted resorcinols suggest that compounds with the 4-substituted resorcinol skeletons exhibit potent tyrosinase inhibitory activity [27]. The additional group, a farnesyl group at position C-3′ was not affected to tyrosinase activity when compared with the activity of apigenin (without farnesyl at position C-3′) [28], while the substitution at position C-3 was critical for the tyrosinase inhibitory activity of the flavone group [29]. Thus, 3-(hydroxyprenyl) isoetin, 3-prenyl-5,7,2′,5′-tetrahydroxy-4′-methoxyflavone, and artocarpin, which were substituted with prenyl, as well as isocycloartobiloxanthone, which was substituted with cyclohexane at position C-3, did not exhibit antityrosinase activity.

However, only artocarpanone could reduce the amount of melanin content and exhibited intracellular antityrosinase activity on B16F1 melanoma cells. This could be due to melanogenesis processes, such as microphthalmia-associated transcription factor (MITF), because melanogenesis-related enzyme expression can activate or depress by up or down regulation of MITF activity; therefore it could exhibit a stimulatory or inhibitory effect on melanogenesis [30,31,32].

3-(Hydroxyprenyl) isoetin, 3-prenyl-5,7,2′,5′-tetrahydroxy-4′-methoxyflavone, isocycloartobiloxanthone, artocarpanone, and artocarpin exhibited potential antibacterial activity against Gram-positive bacteria (*S. epidermidis*, *S. aureus*, MRSA and *C. acnes*), owing to the substituents and positions of hydroxyl and isoprenyl groups. The saturation of the C2=C3 double bond and the presence of hydroxyl groups at C-5, C-6, or C-7 (especially at C-5 and C-7) of the A ring [33,34], as well as another position, such as C-2′ or ### The meaning is fine, just correcting some words.C-4′ of the B ring, could improve the antibacterial effect [35]. Moreover, an increase of hydrophobicity by prenylation could enhance antibacterial activity, especially in substituents with isoprenyl groups at C-6 or C-8 [35]. Furthermore, diprenylated substituents wer more effective against the Gram-positive bacteria than monoprenylated substituents [33].

## 4. Experimantal Section

### 4.1. Plant Materials

Stems of *Artocarpus chama* Buch. Was collected from Southern Literature Botanical Garden, Songkhla Province. The plant was identified by a botanist of Southern Literature Botanical Garden, and the voucher specimen number was SKP 117 01 03 01. The sample specimen was kept at the Department of Pharmacognosy and Pharmaceutical Botany, Faculty of Pharmaceutical Sciences, Prince of Songkla University, Thailand.

### 4.2. Extraction and Isolation

6.8 kg of *A. chama* stem powder was macerated repeatedly with petroleum ether for 3 days (3 times). Then, the extraction was filtrated, and the solvent was evaporated using a rotary evaporator under reduced pressure at 40 °C to yield a petroleum ether extract. Then, the marc was macerated three times with ethyl acetate and methanol for 3 days (3 times each) and boiled with H_2_O. Next, the solvent was removed to provide ethyl acetate, methanol, and H_2_O extracts.

**Scheme 1:**
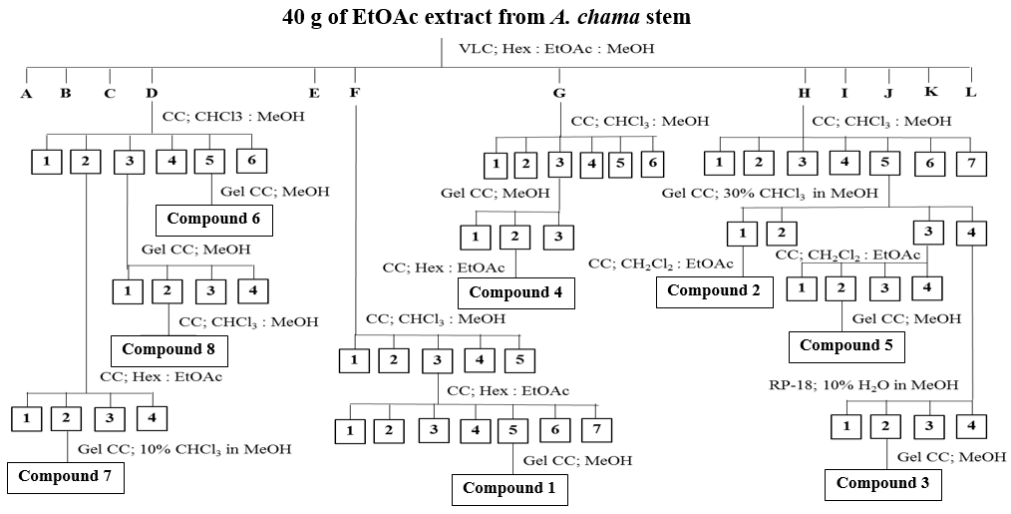
Phytochemical investigation of ethyl acetate extract (EtOAc) from *A. chama* stem. VLC, vacuum liquid chromatography; CC, classical column chromatography; gel CC, Sephadex^®^ LH-20; FA, formic acid; Hex, Hexane; CH_2_Cl_2_, dichloromethane; CHCl_3_, chloroform; EtOAc, ethyl acetate; MeOH, methanol; H_2_O, water.

Based on screening results, the ethyl acetate extract showed potent effects on antityrosinase and antimicrobial activities. Therefore, it was selected for further phytochemical investigation. First, the ethyl acetate extract (40 g) was fractionated by quick-column chromatography, and the eluates were examined by TLC using a gradient solvent system between hexane, ethyl acetate, and methanol. The fractions that showed the similar TLC fingerprints were combined. The interesting fractions, D, F, G and H, were selected to isolate and purify. The isolation step is illustrated in Scheme 1. Afterward, the chemical structures of the isolated compounds were interpreted using nuclear magnetic resonance (NMR) and other spectroscopic techniques.

### 4.3. Spectroscopic Data

#### 4.3.1. 3′-Farnesyl Apigenin; C_30_H_34_O_5_ (Compound **1**)

^1^H-NMR (500 MHz, CD_3_OD): 7.63 (1H, d, *J* = 2.4 Hz, H-2′), 7.62 (1H, dd, *J* = 8.2, 2.4 Hz, H-6′), 6.87 (1H, d, *J* = 8.2 Hz, H-5′), 6.49 (1H, s, H-3), 6.39 (1H, d, *J* = 2.1 Hz, H-8), 6.18 (1H, d, *J* = 2.2 Hz, H-6), 5.34 (1H, td, *J* = 7.3, 1.2 Hz, H-2″), 5.10 (1H, td, *J* = 7.0, 1.2 Hz, H-7″), 4.94 (1H, tt, *J* = 7.0, 1.4 Hz, H-12″), 3.35 (2H, d, *J* = 7.3 Hz, H-1″), 2.14 (2H, dd, *J* = 28.7, 6.5 Hz, H-6″), 2.08 (2H, dd, *J* = 28.5, 6.5 Hz, H-5″), 1.92 (2H, dd, *J* = 33.4, 7.0 Hz, H-11″), 1.85 (2H, dd, *J* = 32.7, 7.0 Hz, H-10″), 1.73 (3H, d, *J* = 1.3 Hz, H-4″), 1.56 (3H, d, *J* = 1.4 Hz, H-14″), 1.54 (3H, d, *J* = 1.3 Hz, H-9″) and 1.48 (3H, d, *J* = 1.3 Hz, H-15″).

^13^C-NMR (125 MHz, CD_3_OD): 183.83 (C-4), 166.57 (C-2), 165.93 (C-7), 163.19 (C-5), 160.50 (C-4′), 159.39 (C-8a), 137.44 (C-3″), 136.15 (C-8″), 131.96 (C-13″), 130.37 (C-3′), 128.92 (C-2′), 126.86 (C-6′), 125.40 (C-12″), 125.14 (C-7″), 123.48 (C-2″), 123.10 (C-1′), 116.25 (C-5′), 105.33 (C-4a), 103.68 (C-3), 100.10 (C-6), 95.05 (C-8), 40.80 (C-10″), 40.72 (C-5″), 28.93 (C-1″), 27.77 (C-11″), 27.37 (C-6″), 25.81 (C-14″), 17.66 (C-15″), 16.26 (C-4″) and 16.16 (C-9″).

#### 4.3.2. 3-(Hydroxyprenyl) Isoetin; C_20_H_18_O_8_ (Compound **2**)

^1^H-NMR (500 MHz, CD_3_OD): 7.13 (1H, s, H-6′), 6.37 (1H, d, *J* = 1.7 Hz, H-8), 6.33 (1H, s, H-3′), 6.16 (1H, d, *J* = 1.9, Hz, H-6), 6.09 (1H, d, *J* = 9.2 Hz, H-9), 5.42 (1H, dt, *J* = 9.2, 1.2 Hz, H-10), 1.92 (3H, d, *J* = 1.2 Hz, H-13) and 1.68 (3H, d, *J* = 0.8 Hz, H-12).

^13^C-NMR (125 MHz, CD_3_OD): 166.40 (C-4′), 165.47 (C-7), 163.31 (C-5), 158.61 (C-8a), 157.61 (C-2), 152.58 (C-2′), 141.95 (C-5′), 139.59 (C-11), 122.45 (C-10), 110.49 (C-1′), 109.94 (C-6′), 107.77 (C-3), 105.64 (C-3′), 105.44 (C-4a), 99.99 (C-6) and 95.01 (C-8), 70.21 (C-9), 25.93 (C-12) and 18.66 (C-13).

#### 4.3.3. 3-Prenyl-5,7,2′,5′-tetrahydroxy-4′-methoxyflavone; C_21_H_20_O_7_ (Compound **3**)

^1^H-NMR (500 MHz, CD_3_OD): 6.71 (1H, s, H-6′), 6.59 (1H, s, H-3′), 6.23 (1H, d, *J* = 2.0 Hz, H-8), 6.17 (1H, d, *J* = 2.0 Hz, H-6), 5.05 (1H, m, H-10), 3.71 (3H, s, 4′-OCH_3_), 3.01 (2H, d, *J* = 7.0 Hz, H-9), 1.58 (3H, d, *J* = 0.5 Hz, H-12) and 1.36 (3H, d, *J* = 0.5 Hz, H-13).

^13^C-NMR (125 MHz, CD_3_OD): 183.53 (C-4), 165.57 (C-7), 163.24 (C-5), 162.97 (C-2), 159.73 (C-8a), 152.53 (C-4′), 149.69 (C-2′), 139.93 (C-5′), 132.68 (C-11), 122.67 (C-10), 121.85 (C-3), 117.83 (C-6′), 113.71 (C-1′), 105.31 (C-4a), 101.43 (C-3′), 99.57 (C-6), 94.46 (C-8), 56.71 (4′-OCH_3_), 24.85 (C-9), 25.82 (C-12) and 17.56 (C-13).

The known natural products, homoeriodictyol (compound **4**) [36], isocycloartobiloxanthone (compound **5**) [37], artocarpanone (compound **6**) [38], naringenin (compound **7**) [39], and artocarpin (compound **8**) [38], were identified by comparing their NMR data with previously published data.

### 4.4. Enzymetic Antityrosinase Activity Assay

Antityrosinase activity was determined by the dopachrome method. l-dopa was used as the substrate. Oxidation of l-Dopa to dopachrome which, represented by red color, could be detected by visible light at 492 nm [40,41]. Briefly, 140 µL of phosphate buffer pH 6.8, 20 µL of sample solution (200 µg/mL), and 2 µL of tyrosinase enzyme solution (203.3 unit/mL) were mixed in a 96-well plate at 25 °C for 10 min. Then, 20 µL of l-Dopa (0.85 mM) was added, and optical density (OD) was detected. After incubation at 25 °C for 20 min, OD was detected again. Dimethyl sulfoxide (DMSO) was used as a negative control. Kojic acid and water extract of *A. lakoocha* wood were used as positive controls. The percent inhibition of tyrosinase reaction was calculated by the following equation:

Tyrosinase inhibition (%) = [1 − (OD_492_ of sample/OD_492_ of control)] × 100.

### 4.5. Cell Culture

Murine melanoma B16-F1 cells (CLS-400122) were cultured in DMEM or Dulbecco’s modified Eagle medium containing 10% FBS heat-inactivated fetal bovine serum at 37 °C in a humidified atmosphere with 5% CO_2_. When cells reached 70–80% confluence cell viability, cellular tyrosinase activity and melanin content were measured [28,42,43,44].

### 4.6. Cell Viability Assay

Cell viability was determined by Sulforhodamine B (SRB) assay. First, 5 × 10^3^ cells/well were seeded in a 96-well plate, incubated for 24 h, and treated with test samples; control cells were treated with 0.5% DMSO. After 48 h incubation, cells were fixed with 10% trichloroacetic acid (TCA) and kept at 4 °C for 1 h. After being strained with 0.45% SRB, 10 mM Tris base was added and shaken to dissolve the SRB color. Optical densities were determined at 492 nm. The percent cell viability was calculated.

### 4.7. Intracellular Antityrosinase Activity and Melanin Content Assays

First, 3 × 10^5^ cells/well were seeded in 12-well plates and allowed to adhere at 37 °C for 12 h. Cells were treated with test samples; control cells were treated with 0.5% DMSO. After 48 h incubation, cells were lysed with RIPA and centrifuged at 14,000 rpm for 20 min (4 °C) in order to separate the supernatant for measurement of tyrosinase activity, and the cell pellet was measured for melanin content.

#### 4.7.1. Intracellular Antityrosinase Activity

The supernatant was collected for determination of protein content by the Bradford method, with bovine serum albumin (BSA) used as a standard. The supernatant of lysate cells and 2 mg/mL l-Dopa were added to a 96-well plate and incubated at 25 °C for 1 h. After that, optical densities were determined at 492 nm. Tyrosinase inhibition was then calculated.

#### 4.7.2. Melanin Content

Cell pellets were dissolved with 1M NaOH and incubated at 55 °C for 1 h. Melanin content was calculated by comparison with the standard curve of synthetic melanin at 475 nm.

### 4.8. Antimicrobial Activity Assay

Microorganisms that cause skin infection, such as *Staphylococcus aureus* (ATTC 25923), *Staphylococcus epidermidis* (TISTR 517), *Cutibacterium acnes* (DMST 14916), and methicillin-resistant *Staphylococcus aureus* (DMST20654),t were selected for this study. The isolated compounds were determined for minimum inhibitory concentration (MIC) and minimum bactericidal concentration (MBC) by a modified broth microdilution method [45,46]. Briefly, 20 µL of the test samples (dilution series ranging from 2560 to 1.25 µg/mL) was added in wells 1 to 12. Then, 80 µL of media was added into each well. Next, 100 µL of the inoculum (concentration 10^6^ CFU/mL) was added in each well. The final concentration of inoculum in each well was 5 × 10^5^ CFU/mL. Then, the cultures were incubated follow the conditions. Incubation mixtures showing positive results of inhibitory effect (MIC) were streaked on media agar, and then incubated by following the conditions. The lowest concentration that did not show any growth was taken as the MBC.

## 5. Conclusions

Three new compounds (**1**–**3**) and five known compounds (**4**–**8**) were isolated from the stem of *A. chama*. The chemical structures of the isolated compounds were identified on the basis of their physical properties and spectroscopic data. Compounds **1**, **4**, and **6** were affected to tyrosinase inhibitory activity, while compounds **3**, **4**, **5**, **6,** and **8** showed potential effects on antimicrobial activity.

This study is the first report on antityrosinase and antimicrobial activities of *A. chama*. Additionally, three new compounds were investigated. Some isolated compounds showed effects on biological activities, which can be further studied on in vivo and in clinical trials for potential use in the treatment of hyperpigmentation and/or infectious diseases caused by microorganisms.

## Figures and Tables

**Table 1:** IC_50_ of isolated compounds from *A. chama* on tyrosinase inhibitory activity.

Compound		IC_50_ (µg/mL)
**1**	3′-farnesyl-apigenin	135.70 ± 1.16
**2**	3-(hydroxyprenyl) isoetin	>200
**3**	3-prenyl-5,7,2′,5′-tetrahydroxy-4′-methoxyflavone	>200
**4**	homoeriodictyol	52.18 ± 0.95
**5**	isocycloartobiloxanthone	>200
**6**	artocarpanone	38.78 ± 1.02
**7**	naringenin	>200
**8**	artocarpin	>200
Std.	Kojic acid ^P^	38.67 ± 0.94
Std.	Water extract of *A. lakoocha* wood ^P^	8.73 ± 0.69

^P^ for positive standards.

**Table 2:** MIC and MBC of isolated compounds against *S. epidermidis*, *S. aureus*, MRSA, and *C. acnes*.

Compounds	*S. epidermidis*		*S. aureus*		MRSA		*C. acnes*	
	MIC (µg/mL)	MBC (µg/mL)	MIC (µg/mL)	MBC (µg/mL)	MIC (µg/mL)	MBC (µg/mL)	MIC (µg/mL)	MBC (µg/mL)
**1**	256	>256	256	>256	>256	>256	>256	>256
**2**	16	32	16	32	16	64	128	>256
**3**	16	64	4	16	4	32	128	>256
**4**	128	256	256	>256	>256	>256	>256	>256
**5**	4	8	4	8	16	64	256	>256
**6**	64	128	64	256	128	>256	32	64
**7**	256	>256	>256	>256	>256	>256	>256	>256
**8**	2	32	2	4	2	2	8	32
Oxa ^P^	0.5	0.5	0.125	0.125	NT	NT	0.5	0.5
Van ^P^	NT	NT	NT	NT	0.125	0.5	NT	NT

^P^ for positive controls; Oxa = Oxacillin; Van = Vancomycin.

## Data Availability

Data sharing is not applicable.
